# The Quacks and the Press

**Published:** 1910-03

**Authors:** E. J. Kehoe

**Affiliations:** Lancet Clinic


THE QUACKS AND THE PRESS.—Much has been said recently about the quack and his methods, but nothing whatever about the quack's chief ally, the press, without the aid of which the schemes of the charlatans would fall flat, as it is the newspaper advertisement that brings the suckers.

One can fill up our daily papers with advertisements so glaringly false and misleading that one can hardly deem it possible that any one of the most ordinary intelligence could be deceived by it; and the only question asked by the management of the paper is, "Can you pay for the space?"

The editors of our papers pose as good citizens, and profess great solicitude in the welfare of their readers, to whom they (the editors) refer as "our fellow citizens." The "fellow citizen" gag, however, does not deter our editorial friends from publishing advertisements which they know are intended as bait to inveigle a lot of these citizens into the clutches of unprincipled rascals.

Let us hope that some day, somewhere, there will be printed a newspaper whose editor will not stoop to the pitiable depths of lending his aid in the cowardly business of robbing the mentally and physically weak, but, on the contrary, will have manhood enough to expose the wolf in sheep's clothing.—*E. J. Kehoe, in Lancet Clinic.*

